# Video compressive sensing for dynamic MRI

**DOI:** 10.1186/1471-2202-13-S1-P183

**Published:** 2012-07-16

**Authors:** Jianing V Shi, Aswin C Sankaranarayanan, Christoph Studer, Richard G Baraniuk

**Affiliations:** 1 Electrical and Computer Engineering, Rice University, Houston, TX 77005, USA

## 

Recent theoretical and practical advances in video compressive sensing (CS) hold considerable promise for the application in dynamic MRI [1,2], an imaging technique for capturing sequence of images. The most profound utility of dynamic MRI is to examine motion, which carries biological signature of malfunctions for various organs, especially the heart and the lung. Dynamic MRI can also examine brain functions, exemplified by perfusion imaging. Compressive sensing enables signal recovery based on number of samples well below Nyquist rate, thereby accelerating the acquisition process of MRI.

Existing literature mainly employs two specific methodologies for dynamic imaging of organs such as the heart and the lung. The first method relies on a set of static imaging acquisitions while the subject holds breathing, and resorts to image registration for retrieving the motion. The second method tries to capture free-breathing motion by using fast imaging techniques, with a trade-off between image resolution and quality. Both methods have their limitations, and we envision video compressive sensing can resolve the challenge.

We propose a novel framework, inspired by a state-of-the-art video compressive sensing algorithm [3], to estimate motion and reconstruct high fidelity dynamic MRI based on partially sampled k-t data. Given highly under-sampled data in the k-t space, we first estimate a low-resolution version of the video at a sequence of sub-sampled time points based on a customized sensing matrix in the Fourier domain. The spatial and temporal resolution of this video is optimized for the best image quality, given the time varying nature of dynamic imaging and the uncertainty principle. Optical flow is consequently estimated between consecutive frames of this reconstructed low-resolution video [4].

Based on the partially sampled Fourier data, and the motion estimate obtained from the optical flow, we use a convex optimization framework to reconstruct the dynamic MRI at full spatial resolution. For the best recovery performance, we minimize an objective function using a mixture of wavelet sparsity of the MRI signal as well as its total variation (TV) in both spatial and temporal dimensions; the objective function is minimized along with two constraints in the least square sense. These constraints (a) enforce consistency of compressive measurements, and (b) impose the estimated optical flow between consecutive frames.

We develop an efficient algorithm to solve for the resultant convex optimization using the alternating direction method based on augmented Lagrangian, further accelerated using Bregman divergence on a sequence of minimization subproblems [5,6].

Our framework enables simultaneous motion extraction and high fidelity video reconstruction in a computational efficient manner, based on highly compressive k-t sampling, therefore making it possible to perform real-time dynamic MRI.
